# Targeting hedgehog-driven mechanisms of drug-resistant cancers

**DOI:** 10.3389/fmolb.2023.1286090

**Published:** 2023-10-23

**Authors:** Jade S. Miller, Natalie E. Bennett, Julie A. Rhoades

**Affiliations:** ^1^ Division of Clinical Pharmacology, Department of Medicine, Vanderbilt University Medical Center, Nashville, TN, United States; ^2^ Pharmacology Training Program, Department of Pharmacology, Vanderbilt University, Nashville, TN, United States; ^3^ Center for Bone Biology, Vanderbilt University Medical Center, Nashville, TN, United States; ^4^ Department of Veterans Affairs, Nashville VA Medical Center, Tennessee Valley Healthcare System, Nashville, TN, United States; ^5^ Program in Cancer Biology, Vanderbilt University School of Medicine, Nashville, TN, United States

**Keywords:** cancer, chemotherapy, resistance, hedgehog, Gli

## Abstract

Due to the cellular plasticity that is inherent to cancer, the acquisition of resistance to therapy remains one of the biggest obstacles to patient care. In many patients, the surviving cancer cell subpopulation goes on to proliferate or metastasize, often as the result of dramatically altered cell signaling and transcriptional pathways. A notable example is the Hedgehog (Hh) signaling pathway, which is a driver of several cancer subtypes and aberrantly activated in a wide range of malignancies in response to therapy. This review will summarize the field’s current understanding of the many roles played by Hh signaling in drug resistance and will include topics such as non-canonical activation of Gli proteins, amplification of genes which promote tolerance to chemotherapy, the use of hedgehog-targeted drugs and tool compounds, and remaining gaps in our knowledge of the transcriptional mechanisms at play.

## 1 Introduction

The Hedgehog (Hh) signaling pathway was first described in 1980, and its roles in embryogenesis and cell polarity have been detailed in countless publications (Van Schmus et al., 1980). Targets of Hh signaling include several cyclins and regulators of mitosis including cyclin D1, cyclin D2, cyclin B, and p21 (Di Magliano and Hebrok, 2003). Several cancers including non-small cell lung cancers (NSCLC), gliomas, and breast carcinomas upregulate Hh signaling, thereby resisting cell cycle arrest (Fan and Khavari, 1999). Furthermore, mutations in the pathway are associated with a hereditary form of basal cell carcinoma (BCC) and overexpression of Shh is sufficient to cause spontaneous skin tumors (Oro et al., 1997; Yang et al., 2010).

Due to its significance in tumorigenesis, a handful of Hh pathway inhibitors have been FDA-approved for treatment of BCC, but both intrinsic and acquired resistance is prevalent due to receptor mutations (Atwood et al., 2015). Mechanisms of resistance to these targeted therapies will be discussed in further detail in Section 2 of this review.

Despite the advent of targeted therapies in cancer treatment, chemotherapies such as paclitaxel, cisplatin, and doxorubicin remain a standard of care for most cancer subtypes. These cytotoxic agents have revolutionized the field of oncology, yet an estimated 80%–90% of cancer-related mortalities are due to chemoresistance (Dan et al., 2020). This is due in par to the selective pressure that is inherent to chemotherapy, as a handful of surviving cells go on to proliferate, resulting in a malignancy that is newly resistant to treatment (Yu et al., 2017a). In this review, we will discuss the ways in which Hh signaling activates transcriptional programs which enable cancer cells to evade chemotherapy and targeted cancer therapies (summarized in Table 1).

**TABLE 1:** Summary of known Hh pathway-mediated mechanisms of anticancer drug resistance.

	Cancer type	Therapy	References
Induction of EMT			
	Non-small cell lung cancer	Gefitinib (EGFRi)	Chen et al. (2022)
	Colorectal cancer	5-Fluorouracil	Zhang et al. (2017)
	Bladder cancer	Gemcitabine	Amantini et al. (2016)
	Bladder cancer	Doxorubicin	Amantini et al. (2016)
	Breast cancer		Riaz et al. (2019)
	Pancreatic cancer		Wang et al. (2016)
	Cervical cancer		Sharma et al. (2019)
Smo and EGFR-driven mechanisms			
*SMO* gene amplification	Non-small-cell lung cancer	Gefitinib	Della Corte et al. (2015)
		Erlotinib	
		Cisplatin	
*SMO* point mutations	Basal cell carcinoma	Vismodegib	Atwood et al. (2015)
Synergism with EGFR	Non-small-cell lung cancer	Gefitinib	Bai et al. (2016)
	Basal cell carcinoma		Eberl et al. (2012)
Transcriptional upregulation of *SOX2*	Pancreatic ductal adenocarcinoma	Gemcitabine	Jia et al. (2019)
Transcriptional upregulation of *TAP1*	Hepatocellular carcinoma	Sorafenib	Zhou et al. (2020)
		Doxorubicin	
		Cisplatin	
Deacetylation of Gli2 by SIRT1	Multiple myeloma	Bortezomib	Xie et al. (2019)
Alteration of Ca^2+^ homeostasis	Lung adenocarcinoma	Cisplatin	Tian et al. (2012)
Activation of Bcl-2	Multiple myeloma	Bortezomib	Liu et al. (2014)
		Melphalan	
	Glioma	Temozolomide	Cui et al. (2010)
Inducible glucuronidation of chemotherapy	Acute myeloid leukemia	cytarabine	Zahreddine et al. (2014)
Disruption of tumor-stromal crosstalk	Pancreatic adenocarcinoma	Gemcitabine	Khan et al. (2020)
	Breast cancer	Docetaxel	Cazet et al. (2018)
Transcriptional upregulation of ABC transporters			
ABCB1	Acute Myeloid Leukemia	Doxorubicin	Queiroz et al. (2010)
		Mitoxantrone	
		Vincristine	
	Breast cancer	Doxorubicin	Chen et al. (2012)
	Colorectal cancer	5-Fluorouracil	Po et al. (2020)
		Oxaliplatin	
	Esophageal adenocarcinoma	Docetaxel	Sims-Mourtada et al. (2007)
	Prostate cancer		
	Glioma	Temozolomide	Cui et al. (2010)
	Rhabdomyosarcoma	Vincristine	Yoon et al. (2020)
	Ewing’s sarcoma		
	Ovarian	Cisplatin	Steg et al. (2012), Song et al. (2018), Zhang et al. (2020a), Chen et al. (2021), Wang et al. (2022)
	Non-small cell lung cancer	Mitoxantrone	Zhang et al. (2022)
		Daunorubicin	
ABCG2	Esophageal adenocarcinoma	Docetaxel	Sims-Mourtada et al. (2007)
		Etoposide	
		Methotrexate	
	Gastric cancer	5-Fluorouracil	Yu et al. (2017a)
	Non-small cell lung cancer	Mitoxantrone	Zhang et al. (2022)
		Daunorubicin	
	Prostate cancer	Docetaxel	Sims-Mourtada et al. (2007)
ABCC1	Hepatocellular carcinoma	Itraconazole	Ding et al. (2017)
		Sonidegib	
	Glioma	Temozolomide	Cui et al. (2010)
Dysregulation of DNA repair			
Downregulation of MGMT	Glioma	Temozolomide	Cui et al. (2010), Li et al. (2016), Wang et al. (2017)
Upregulation of NBS1	Colorectal cancer	5-Fluorouracil	Zhang et al. (2020b)
Upregulation of γ-H2AX	Osteosarcoma	Cisplatin	Chen et al. (2021)
	Pancreatic cancer	Radiation	Wu et al. (2013)
	Colorectal cancer		Mazumdar et al. (2011), Agyeman et al. (2012)
Upregulation of ERCC1, XPD, XRCC1	Ovarian cancer	Cisplatin	Kudo et al. (2012)
Downregulation of MLH1	Pancreatic cancer	Methylnitrosourea	Inaguma et al. (2013)

### 1.1 The canonical hedgehog signaling pathway

There are three ligands present in vertebrates which may activate the Hh signaling pathway: Sonic hedgehog (Shh), Indian hedgehog (Ihh), and Desert hedgehog (Dhh). In normal tissues, Dhh is primarily expressed in mammals during gonad development, Ihh is expressed in chondrocytes during endochondral ossification, and Shh is expressed in many developing tissues to regulate processes such as limb development, neural patterning, and proximal-distal patterning (Van Schmus et al., 1980; Vortkamp et al., 1996). Shh is also the ligand which is most often implicated in malignancies.

The Hh signaling pathway begins at the primary cilia (PC), a structure that protrudes from the cell and is formed by microtubules. The target for Hh ligands is Patched (encoded by the gene PTCH1), a transmembrane receptor which suppresses Smoothened (Smo) in the absence of ligand, preventing activation of downstream effectors of Hh signaling. Loss-of-function mutations in *PTCH1* result in ligand-independent activation of SMO and constitutive Hh signaling, and these mutations are often a driver of malignancy. *PTCH1* mutations are found in as many as 73% of basal cell carcinomas and are also associated with development of medulloblastoma and recurrence of breast cancer (Zurawel et al., 2000; Bonilla et al., 2016; Wang et al., 2019).

**FIGURE 1:**
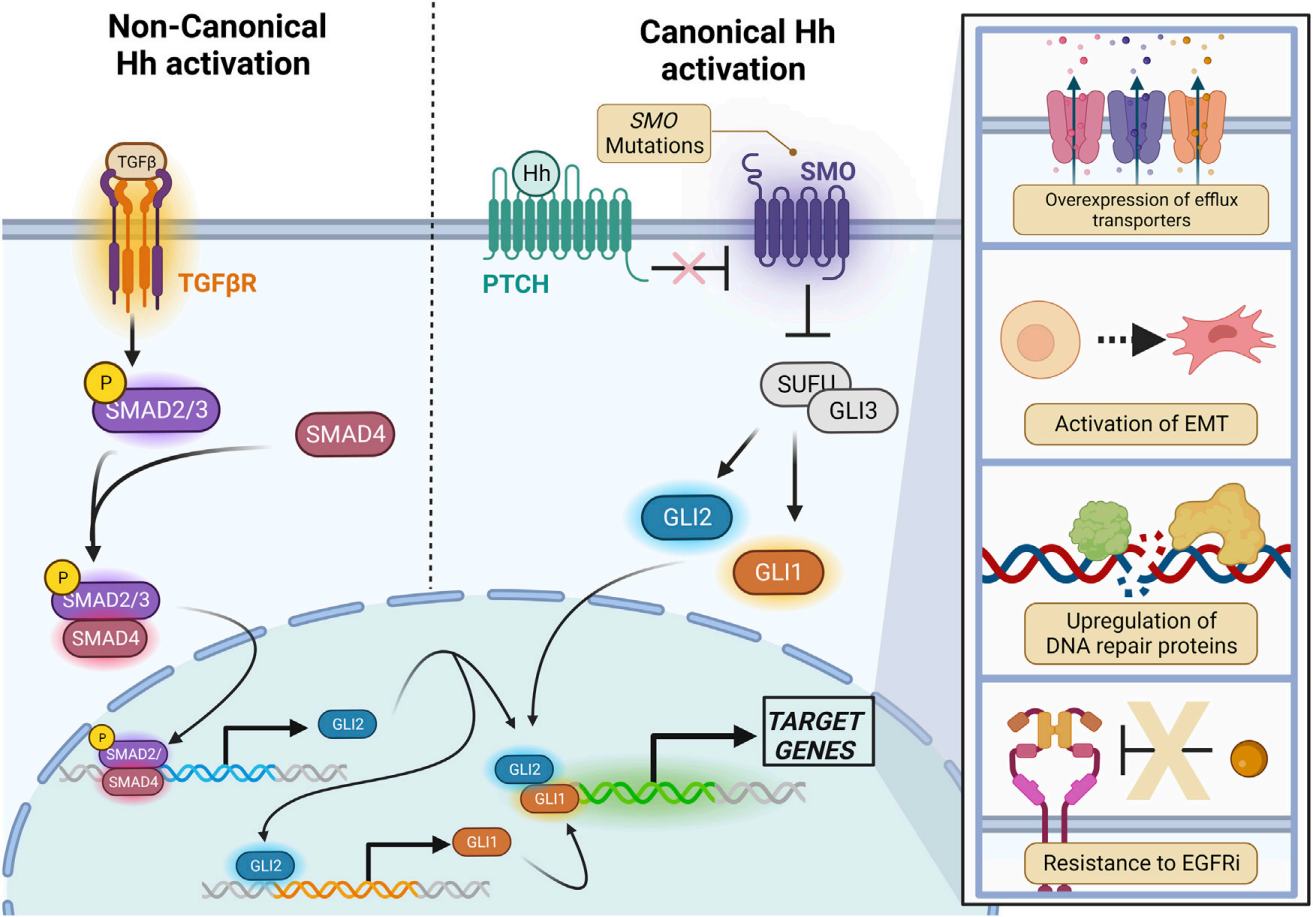
Canonical and Non-canonical Hh signaling converges on Gli proteins. Canonical (Smo-dependent) signaling is the result of a Hh ligand binding to Ptch, relieving its inhibition on Smo. Upon Smo activation, SUFU is inhibited, releasing the Gli1 and Gli2 transcription factors which then translocate into the nucleus to activate transcriptional targets. Gli2 may also be non-canonically activated by SMAD2/SMAD4 downstream of TGF-β. Created with BioRender.

In the presence of ligand, the 7-transmembrane receptor SMO (discussed in section 2.1) is phosphorylated and translocates into the PC where it inhibits Suppressor of Fused (SUFU), releasing the Glioma-associated oncogene (Gli) proteins Gli1 and Gli2. The Gli proteins localize to the nucleus upon their activation, where they go on to bind to their transcriptional targets (Figure 1).

Each of the Gli transcription factors (Gli1, Gli2, and Gli3) activate a diverse subset of genes, including several overlapping targets. Gli1 was the first discovered and is perhaps the most extensively characterized in the field of developmental biology, with high expression in mesenchymal stem cells (MSCs), neural stem cells (NSCs), and embryonic stem cells (ESCs) (Kinzler and Vogelstein, 1990). Gli1 and Gli2 share many transcriptional targets, and Gli2 can itself transcriptionally amplify Gli1 (Ali et al., 2019; Avery et al., 2021). Transcriptional targets of Gli2 also include NF-κB1, SMAD2, and Runx2 (Ali et al., 2019). In contrast to Gli1 and Gli2, Gli3 is active in the *absence* of Hh activation, and acts as a transcriptional repressor of Gli1 and Gli2 targets (Matissek and Elsawa, 2020).

The Gli transcription factors are also regulated through proteolytic processing. The *GLI1* mRNA transcript may be alternatively spliced to generate an N-terminal deletion (GLI1ΔN) and a truncated variant (tGLI1), which are loss-of-function and gain-of-function mutations, respectively (Doheny et al., 2020). Unlike the other isoforms, tGli1 is expressed only in cancerous tissues and promotes angiogenesis in glioblastoma and breast cancer brain metastases (Doheny et al., 2020). Both Gli2 and Gli3 possess a processing determinant domain (PDD), and variations in this region contribute to differential proteolytic cleavage; Gli3 is proteolytically processed to a much greater extent than Gli2 *in vivo* (Pan and Wang, 2007). These cleaved forms of Gli2 and Gli3 act as transcriptional repressors of Gli1 and Gli2 targets and negatively regulate response to Hh signaling (Li et al., 2011; Matissek and Elsawa, 2020). Additionally, there are two other isoforms of Gli2 found in humans which may be differentially expressed in malignancy, but the functions of these variants are not well understood (Tojo et al., 2003).

There is some debate on whether drug resistance mechanisms are driven by Gli1 or Gli2. While the literature has indicated the importance of both transcription factors, it should be noted that Gli1 is a transcriptional target of Gli2, as overexpression of Gli2 results in upregulation of both Gli2 and Gli1, but the reverse is not true (Agyeman et al., 2012). Additionally, Gli2, but not Gli1, is required for aberrant activation of Hh signaling (Bai et al., 2002). Co-amplification of Gli1 and Gli2 occurs frequently in malignancy, but dependence on Gli1 or Gli2 varies between cancers, between subtypes, and between cell lines of the same subtype; Hh may even be activated heterogenously within the tumor microenvironment (Giammona et al., 2023). There are a multitude of publications indicating the importance of Hh signaling in the tumor immune microenvironment, from polarization of macrophages to T cell differentiation and immunosuppression, but the relationship between Hh signaling, cancer immunity, and resistance to therapy is still being established (Wang et al., 2023).

### 1.2 Non-canonical Hh signaling

Overexpression of Gli1 or Gli2 is observed in a wide array of chemoresistant malignancies, and this increase is often not accompanied by an increase in Shh ligands or Smo expression (Yu et al., 2017b). This is because Hh signaling may be activated independently of Smo as the result of several signaling cascades, most notably by Transforming growth factor β (TGF- β) (see below), or Epidermal growth factor (EGF) (discussed in Section 2.2) (Pietrobono et al., 2019).

#### 1.2.1 Non-canonical Hh activation by TGF- β

Transforming Growth Factor-β (TGF-β) signals through the serine/threonine kinase receptors TGFbeta-RI and TGFbeta-RII, which both exist as dimers in their unbound form. Ligand binding to TGFbeta-RII induces its dimerization with TGFbeta-RI, forming a tetramer. The activated tetramer is autophosphorylated and recruits and phosphorylates receptor-regulated SMADs (r-SMADs) such as SMAD2 and SMAD3, which dissociate from the receptor complex and bind common mediator SMADs (co-SMADs) including SMAD4. The r-SMAD/co-SMAD complex translocates to the nucleus, inducing transcription of TGF-β-target genes, one of which is *GLI2* (Dennler et al., 2007).

When discussing Hh-driven drug resistance, it is also important to acknowledge the role of non-canonical Hh signaling in response to anticancer therapies. TGF-β is often overexpressed in malignancy and its amplification has been linked to resistance to chemotherapy, immunotherapy, and targeted therapies (Zhang et al., 2021). Interestingly, large-scale transcriptomic analysis of 37 cancers has revealed a tighter correlation between *TGFB* and G*LI2* gene expression than between *SHH* and *GLI2* for prediction of an epithelial mesenchymal transition signature (de Reyniès et al., 2020). Additionally, activation of Gli2 and Gli2 is dependent on SMAD3 in several cell lines including keratinocytes and breast carcinomas, and activation of the Gli2 target gene *PTHLH* (encoding parathyroid hormone-related protein) occurs independently of canonical Hh signaling (Dennler et al., 2007; Johnson et al., 2011). In fact, TGF-β2 is itself a target of Gli2, indicating the presence of a positive feedback loop between the TGF-β and Hh signaling pathways (Javelaud et al., 2012; Xu et al., 2019). However, the molecular mechanisms and protein-protein interactions of TGF-β/Hh crosstalk are not fully understood.

### 1.3 Hedgehog-mediated activation of EMT

The process of epithelial to mesenchymal transition (EMT) occurs in healthy tissues during embryogenesis, allowing migration of mesenchymal cells through epithelial boundaries which would normally restrain them. This occurs through downregulation of adhesion proteins, separation of cell-cell junctions, cytoskeletal rearrangement, and secretion of matrix metalloproteinases (MMPs) which degrade the basement membrane, enabling movement through layers of tissue (Chen et al., 2011).

In the context of malignancy, cancer cells may undergo EMT in order to proliferate, invade, and metastasize. Cancers downregulate E-cadherin in favor of N-cadherin, which tethers with a lower affinity to the extracellular matrix (Their, 2002). This is accompanied by expression of transcription factors including Snail, Slug, ZEB1, ZEB2, and Twist as the result of enhanced growth factor signaling pathways including fibroblast growth factor (FGF) and TGF-β (Xu et al., 2009). Collectively, these transcription factors function to suppress apoptosis, prevent senescence, promote survival, and in some cases, confer resistance to a wide range of anticancer therapies.

One of the earliest observations of the correlation between EMT and chemoresistance was published in 1992 when Sommers et al. (1992) observed elevated expression of vimentin (a mesenchymal marker) in both Doxorubicin-resistant and Vinblastine-resistant breast cancer cell lines, correlating with enhanced invasive capacity. EMT may also drive resistance to targeted therapies such as EGFR inhibitors, as genetic silencing of *TWIST1* sensitized EGFR-mutant lung cancer to osimertinib and erlotinib (Yochum et al., 2018).

While Hh signaling is certainly not the only mechanism in common between EMT and drug resistance, there are several studies in which Hh signaling has been implicated in cancer stemness or a mesenchymal phenotype. For example, Gli1 induction of Snail, nuclear localization of β-catenin and subsequent EMT has been documented in several cancers including ovarian, breast, and basal cell carcinoma (Riaz et al., 2019). Canonical Hh signaling also appears to drive a cancer stem-like phenotype in pancreatic cancer, as Smo knockdown resulted in reduction of EMT, self-renewal, and chemoresistance to gemcitabine (Wang et al., 2016). Non-canonical activation of Hh signaling is also a key component of EMT, as TGF-β signaling is frequently activated in the mesenchymal state (Xu et al., 2009). Interestingly, there appears to be a positive feedback loop between EMT and activation of Hh; loss of E-cadherin results in elevated expression of Shh, Ptch, Smo, Gli1, and Gli2. Conversely, pharmacological inhibition of Gli transcription results in reduction of sphere formation and invasive capacity (Sharma et al., 2019).

## 2 The role of hedgehog signaling in resistance to targeted therapy

Because Hh signaling activates such a broad range of transcriptional programs, it is a key mediator of resistance to targeted therapies. This section of the review will focus on the two drug classes whose resistance mechanisms are the most relevant to Hh signaling: Smo inhibitors and EGFR inhibitors.

### 2.1 Resistance to Smo inhibitors

There are currently three Smo inhibitors which have received FDA approval; Vismodegib (GDC-0449, Erivedge^®^) and sonidegib (ODOMZO^®^) were FDA-approved in 2012 and 2015, respectively, for treatment of patients with metastatic or locally advanced BCC. Additionally, Glasdegib (Daurismo^®^) is FDA-approved for the treatment of acute myeloid leukemia in combination with low-dose cytarabine (Nguyen and Cho, 2022). However, objective response rate to these therapies (47.6%, 38%, and 26.9%, respectively) remain hindered by both primary resistance (an absence of response to therapy) and secondary resistance (initial response followed by acquisition of resistance) (Cortes et al., 2019).

Most mutations in *SMO* fall into one of two categories. Mutations which occur in the ligand binding pocket disrupt chemical interactions with Smo inhibitors such as vismodegib. Alternatively, mutations may occur within the transmembrane helices of the receptor, resulting in constitutive activity and consequently, constitutive Gli activation. These variants often arise as the result of targeted therapy. Exome sequencing has revealed that as many as 50% of resistant basal cell carcinomas contain at least one mutation in *SMO* (Atwood et al., 2015). Mutations in other genes of the Hh pathway (including *PTCH* or *SUFU*), or amplification of Gli1 or Gli2 often occur independently of Smo, generating primary resistance to this drug class.

Fortunately, there may be a way to overcome these occurrences. There is overwhelming evidence (some of which is summarized in this review) that Gli inhibition may be an effective strategy for the evasion of Smo inhibitor-resistant cancers (Nguyen and Cho, 2022). Such Gli inhibitors do exist but are primarily in use as tool compounds with minimal translational or pre-clinical usage, highlighting a need for specific, potent inhibitors which function downstream of Smo.

### 2.2 Hedgehog activation in TKI resistance

Tyrosine kinase inhibitors (TKIs) such as gefitinib, erlotinib, and cabozantinib are small-molecule compounds which specifically inhibit the activity of receptor tyrosine kinases such as the epidermal growth factor receptor (EGFR), vascular endothelial growth factor (VEGFR) and platelet-derived growth factor receptor (PDGFR). However, prolonged exposure to these targeted therapies frequently results in resistance and relapse (Raez et al., 2022).

Surprisingly, one mechanism for resistance to TKIs may be amplification of Hh signaling. Cooperativity between downstream effectors of EGFR and Hh signaling was first observed in stem cells of the mammalian neocortex, and further evidence for EGFR-Hh synergism was presented in transcriptional profiling of immortalized keratinocytes, as EGF signaling modulated transcription of a subset of Gli target genes at the promoter level (Palma and Ruiz i Altaba, 2004; Kasper et al., 2023). This subset included pro-tumorigenic and metastatic markers such as JUN, FGF19, and CXCR4. Furthermore, an *in vivo* model of Gli2-driven basal cell carcinoma, EGFR deletion inhibited growth of Hh-driven lesions. This same study imitated these findings in a xenograft model of pancreatic cancer, which raises questions on the role of EGFR-Hh cooperativity in other malignancies with aberrantly activated Hh signaling (Eberl et al., 2012).

Beyond the synergism observed between EGFR and Hh downstream effectors, evidence also suggests that Hh signaling may itself promote EGFR activation through an MMP-mediated mechanism. Matrix metalloproteinases (MMPs) including MMP-2, MMP-7, and MMP-9 are known transcriptional targets of Hh signaling (Chen et al., 2013; Zhang et al., 2019). Upon their expression, MMPs are secreted into the microenvironment and proteolytically cleave targets in the extracellular matrix, promoting invasion and metastasis. These targets also include ligands such as heparin-binding EGF, or EGF precursors on the cell surface, resulting in ligand-dependent activation of EGFR (Reinchisi et al., 2013).

Currently, the EGFR inhibitor gefitinib is a first-line intervention for patients with advanced non-small cell lung cancer (NSCLC), but a prevalent problem is the occurrence of TKI resistance (Raez et al., 2022). There is considerable evidence for a Hh-driven mechanism of TKI resistance; high plasma levels of Shh in NSCLC patients with advanced disease correlates with poor objective response to several TKI therapies and a reduction in progression-free survival (210 vs. 342 days) (Takam Kamga et al., 2021). These outcomes have been explained by *in vitro* studies in which stimulation with exogenous Shh resulted in increased tolerance to Gefitinib in the NSCLC cell lines A549, PC9, and H1975. Additionally, gefitinib and the Smo antagonist SANT-1 synergistically inhibited proliferation and decreased expression of EMT markers such as E-Cad and Snail (Bai et al., 2016). Similarly, gefitinib and vismodegib (an FDA-approved Smo inhibitor) synergistically reduced NSLC tumors in mice in comparison to either drug as a monotherapy (Chen et al., 2022). Altogether, these studies make a promising argument for cooperativity between EGF and Hh and may inform the optimization of combined TKI and Smo inhibition in the clinic.

Interestingly, in their studies with gefitinib and SANT-1, Bai et al. (2016) observed increased expression of the efflux transporter ABCG2 as the result of exposure to Shh, and diminished ABCG2 after Hh inhibition in TKI-resistant cell lines. While transporter-mediated resistance to TKI is not extensively studied, gefitinib is a known inhibitor of ABCG2, and ABCG2 is a known transcriptional target of Hh signaling (Kitazaki et al., 2005). This implies that the role of Hh in gefitinib resistance is two-fold; first, downstream effectors of Hh signaling are upregulated to compensate for inhibition of EGFR, and second, Hh signaling is one potential mechanism for elevated drug efflux from the cell. The role of the Gli proteins in transcriptional regulation of drug efflux transporters such as ABCG2 will be discussed in further detail later in this review.

## 3 Gli-mediated transcription of ABC transporters

Another mechanism by which cancers acquire resistance is through elevated expression of transmembrane protein pumps known as drug efflux transporters, also known as drug efflux transporters, which results in multidrug resistance (MDR). The transporter family that is most often indicated in malignancy is the ATP-binding cassette (ABC) transporter family. In healthy tissues, ABC transporters serve a wide range of functions including ATP-mediated transport of peptides, xenobiotics, metabolites, and ions, and are expressed intracellularly or extracellularly depending on subfamily. Malignancies most often acquire MDR through transcriptionally upregulating expression of these transporters, preventing chemotherapy from reaching therapeutic concentrations intracellularly (Fletcher et al., 2010).

Drug transporter expression in resistant cell lines varies depending on the mechanism of transport for a given drug molecule. For example, paclitaxel is a known substrate of ABCB1 and ABCC3, as indicated by upregulation of these transporters in PTX-resistant cell lines (Němcová-Fürstová et al., 2016). Differential expression of ABC transporters also depends on subset of malignancy; for example, the HER2+ breast cancer cell line SK-BR-3 upregulates a subset of ABC transporters which is slightly different from MCF-7 (Luminal A) in response to PTX (Němcová-Fürstová et al., 2016).

### 3.1 ABCB1/MDR1/p-GP

The *ABCB1* gene encodes for an efflux transporter protein known as p-Glycoprotein (also known as multidrug resistance gene 1, *MDR1*). ABCB1 is broadly expressed including in adrenal tissue, the gastrointestinal tract, and the liver, and is polyspecific for hundreds of hydrophobic endogenous and exogenous compounds, including a large proportion of chemotherapeutics (Uhlén et al., 2015). Notable substrates include doxorubicin and other anthracyclines, vinblastine, and taxol analogs such as paclitaxel and docetaxel (Robinson and Tiriveedhi, 2020). Therefore, ABCB1 is one of the most clinically significant efflux transporters which play a role in multidrug resistance.

The majority of research on the Gli-mediated transcription of *ABCB1* has been done in models of ovarian cancer. Even between ovarian cancer cell lines, evidence exists for both Gli1 and Gli2-driven mechanisms, and it is unclear whether one or both are required for expression of ABCB1. In a 2014 study, a 34 base-pair segment of the *ABCB1* promoter which contains a Gli-recognition site was cloned into a luciferase reporter vector and transfected into OVCAR3 and A2780 cell lines. Using electrophoretic mobility shift assays (EMSAs), the authors observed displacement of a DNA probe from the Gli-recognition site, which was reversed by the addition of an anti-Gli1 antibody, suggesting that Gli1 may directly bind to *ABCB1*, although this occurred to a greater extent with *ABCG2* (see below) (Chen et al., 2014).

Some conflicting evidence was acquired in SKOV3 ovarian cancer cell lines, as transfection with Gli1 overexpressing plasmid did not result in an increase in *ABCB1* mRNA (Zhang et al., 2020a; Zhang et al., 2020b). In addition to Gli1, a putative binding site for Gli2 was isolated in the promoter of *ABCB1* by Wang et al. using point mutagenesis. This binding sequence is located at −875 to −867 of the *ABCB1* promoter, and mutation resulted in significant reduction of luciferase reporter activity (Wang et al., 2022).

### 3.2 ABCG2/BCRP

ABCG2 (also known as breast cancer resistance protein, BCRP) is an efflux transporter which is most highly expressed in the GI tract, liver, mammary tissue, as well as in both male and female sex organs (Uhlén et al., 2015). Its expression in the liver canalicular membranes also contributes to its protective role against xenobiotics and toxic metabolites. Its substrates include several anticancer drugs including anthracyclines, camptothecin analogs, TKIs, and antimetabolites (Mo and Zhang, 2012).

Generation of 5FU-resistant gastric cancer cell lines resulted in a ∼3-fold increase in *ABCG2* mRNA which was almost completely reversed by *GLI2* shRNA knockdown (Yu et al., 2017b). Similarly, *GLI1* knockdown resulted in significant downregulation of ABCG2 in OVCAR3 and A2780 ovarian cancer cell lines (Chen et al., 2014).

Recently, Hh-mediated ABCG2 expression has been linked to RUNX1, which is a downstream effector of TGF- β and bone morphogenetic protein (BMP) signaling. RUNX1 overexpression conferred resistance to 5-FU in three cell lines of colorectal cancer, which additionally correlated with expression of ABCG2 (Li et al., 2021). RUNX1 has been implicated in the resistance mechanisms of several cancer types including paclitaxel-resistant breast cancer and castration-resistant prostate cancer (Li et al., 2021; Fernández et al., 2023).

### 3.3 ABCC1/MRP1

ABCC1 (also known as Multidrug Resistance Protein 1, MRP1) is expressed nearly ubiquitously in epithelial and endothelial cells of the GI tract, gallbladder, kidney tubules, sex organs, and soft tissues (Uhlén et al., 2015). While there are a considerable number of studies indicating a correlation between Hh signaling and ABCB1 or ABCG2 expression, little work has been done on the relationship between Hh and ABCC1. A study using a subpopulation of stemlike Huh-7 hepatoma cell lines found elevation of Gli2 and ABCC1 in comparison to parental cell lines. Additionally, Gli2 expression correlated with mRNA levels of ABCC1 after pharmacological Smo inhibition (Sonidegib). Notably, Smo inhibition only partially reduced ABCC1 expression indicating the presence of non-canonical Gli activation (Ding et al., 2017). In this particular model of drug resistance, Gli2 did not correlate with ABCB1, ABCG2, or Gli1 expression even though this has been previously described in the literature.

### 3.4 Other ABC transporters

While ABCB1 and ABCG2 are the most studied ABC transporters in the context of chemoresistance, there are many other members of this superfamily which remain unexplored. A screen of protein expression revealed significant reductions in these two transporters after shGli2 knockdown, but significant changes were also observed in ABCA2, ABCB4, ABCC2, ABCC3, ABCC11, and ABCG1. And, somewhat paradoxically, a handful of ABC transporters were upregulated after Gli1 knockdown (Po et al., 2020). Of course, it would be a huge undertaking to study each of these proteins in the context of Hh signaling, but as the field progresses it should be noted that it will be highly unlikely that a unifying model of Gli-mediated efflux transporter expression emerges.

A major obstacle to overcome is the lack of ABC transporter-specific pharmacological inhibitors. In the 1990s and 2000s, clinical trials of inhibitors such as verapamil were terminated as the result of severe adverse events due to the presence of ABC transporters or structurally similar transmembrane pumps in processes such as calcium transport, action potentials, and clearance of metabolites (Stefan, 2019). Because the pumps themselves remain an elusive (if not impossible) drug target, an alternative approach may be the development of drugs which target the tissue or cancer-specific transcriptional mechanisms which result in their expression. Smo inhibitors such as sonidegib and vismodegib have yielded promising results as transcriptional inhibitors of ABCB1 and ABCG2, increasing uptake of substrates such as colchicine and daunorubicin (Zhang et al., 2009; Zhang et al., 2022). However, other potential drug targets in the Hh signaling pathway may reveal themselves as the transcriptional mechanisms of efflux transporter genes are resolved.

## 4 Gli-mediated upregulation of DNA repair proteins

Many chemotherapies incur DNA damage, which at therapeutic doses results in enough accumulation of damage to induce apoptosis. This may occur through formation of crosslinks between DNA strands (platinum agents), topoisomerase inhibition (doxorubicin and other anthracyclines), or formation of covalently bonded DNA adducts (temozolomide and other alkylating agents). Cancer cells often avoid the cytotoxic effects of these drugs through upregulation of efflux transporters (mentioned previously) or by overexpression of proteins involved in DNA damage repair so that DNA replication and mitosis may proceed (Meng et al., 2015).

There is growing evidence that non-canonical Hh signaling (and not Smo-dependent) is a key mechanism by which cancers activate the DNA damage response in order to evade chemotherapy. Upon treatment with cisplatin, ovarian cancer cell lines upregulated proteins associated with nucleotide excision repair, including ERCC1, XPD helicase, and XRCC1, a response which was blocked by shRNA knockdown of Gli1. Interestingly, treatment with cyclopamine had no such effect, suggesting that this particular DNA repair pathway activation occurs independently of canonical Hh signaling (Kudo et al., 2012).

Further evidence for the importance of non-canonical Gli activation was presented by Mazumdar et al. (2011) as the Gli antagonist GANT61, but not cyclopamine, induced growth arrest at G1-S phase and apoptosis. GANT61 treatment induced phosphorylation of H2AX (γH2AX), an early marker of the DNA damage response, as well as phosphorylation of ATM and its effector kinase Chk2, indicating the presence of double-stranded DNA breaks. Despite apparent activation of these proteins, COMET assay revealed significant DNA damage in HT29 cells treated with GANT61 in comparison to cyclopamine or DMSO control, indicating that either Gli1 or Gli2 partially inhibits the DNA damage response. Similar results were achieved by genetic overexpression of Gli3R, which has an inhibitory effect on Gli1 and Gli2 (Mazumdar et al., 2011). Furthermore, GANT61 treatment results in downregulation of genes involved in DNA replication such as thymidylate synthase, thymidine kinase, topoisomerase2, E2F, and DNA polymerase epsilon (Shi et al., 2010). GANT61 also displays synergistic lethality in ovarian cancer cells when combined with the Poly (ADP-ribose) polymerase (PARP) inhibitor Olaparib (Mani et al., 2021).

### 4.1 Regulation of MGMT

O^6^-methylguanine-DNA methyltransferase (MGMT) is a DNA repair enzyme which repairs methylated nucleotides and is often upregulated in response to alkylating agents. Hedgehog-mediated regulation of MGMT has been observed in gliomas which acquire resistance to temozolomide (TMZ), a first-line alkylating chemotherapeutic. In patient glioblastomas, MGMT expression is inversely correlated with response to TMZ therapy. Furthermore, Gli1 expression is negatively associated with prognosis and Gli1 nuclear localization correlates with MGMT expression (Wang et al., 2017).

Overexpression of Gli1 in glioblastoma cell lines results in increased tolerance to TMZ, whereas pharmacological inhibition of Hh signaling using GANT61 and cyclopamine resensitizes cells to chemotherapy, and treatment with cyclopamine reduced tumor burden *in vivo* (Li et al., 2016; Wang et al., 2017). In both cases, MGMT expression was reduced in a dose-dependent manner, suggesting that Hh signaling regulates MGMT expression in some way. However, Li et al. acknowledged in their research that GANT61 inhibits both Gli1 and Gli2. Bioinformatic analysis and chromatin immunoprecipitation of the MGMT promoter revealed a Gli1 recognition site, so while there is strong evidence for Gli1-driven expression of MGMT, whether this mechanism is Gli1 or Gli2-driven has not been fully explored.

## 5 Bone metastases: a potential case of Gli-mediated chemoresistance

The bone microenvironment is fertile soil for metastases; in the case of breast cancer, 60%–70% of patients have skeletal lesions at time of death (Coleman and Rubens, 1987). This is partially due to the presence of growth factors such as TGF-β and IGF, which are released from the bone matrix during bone turnover. This results in aberrant activation of Hh signaling in cancer cells: Gli2 is significantly overexpressed in bone metastases in comparison to healthy tissues and the primary site (Vanderburgh et al., 2019). A key transcriptional target of Gli2 is *PTHLH*, the gene which encodes Parathyroid Hormone-related Protein (PTHrP), an osteolytic factor which is secreted by cancer upon metastasis to the bone. Thus, Hh signaling is a significant driver of tumor-induced osteolytic bone destruction (Sterling et al., 2006).

There is a logical connection between metastasis and multidrug resistance; both metastatic and drug-resistant cancer are marked by a mesenchymal phenotype (see Section 1.3) and the ability to dramatically change their transcriptional programming. Clinical data has shown that when metastases acquire resistance to first-line chemotherapy, switching to an alternative chemotherapy does not improve overall survival (Smerage et al., 2014).

What if intrinsic resistance to chemotherapy is a byproduct of non-canonical Hh activation in these bone metastases? As described above, the transcriptional targets of Gli2 include several ABC transporters which are involved in chemoresistance (Po et al., 2020). Included in these transporters are ABCB1 and ABCC2, which were associated with increased risk of bone metastasis in a cohort of 73 patients (Sensorn et al., 2016). Thus, it may not be surprising that breast cancer cells grown on 3D bone-mimetic scaffolds display increased tolerance to chemotherapy (Kar et al., 2020).

While there is circumstantial evidence to support the idea of Gli-driven chemoresistance in bone metastases, to date no studies have explicitly detailed the molecular interactions which result in this phenotype. However, this is just one of many cases of aberrant Hh activation which merits further study.

## 6 Conclusion

There is a large body of *in vitro* and pre-clinical research on the function of Hh signaling in drug resistance, but due to the heterogeneity of cancer, there is most likely not a unifying model. Smo inhibitors have yielded promising results in clinical practice, but there are still several remaining obstacles to the therapeutic use of Hh inhibition.

A remaining problem with the use of Hh inhibitors to overcome resistance is that in their current form, they themselves are prone to resistance. The use of structural biology and genomics has illuminated the prevalence of polymorphisms in both *SMO* and *PTCH1* which interfere with the use of Smo inhibitors in the clinic. Additionally, there is a growing body of evidence that Hh activation often occurs independently of its canonical receptors, which means that if Hh is to be targeted broadly in cancer therapy, there must be a focus on the Hh pathway downstream of Smo. Gli inhibitors such as GANT58 and GANT61 have the potential to sensitize a broad range of malignancies to several targeted and cytotoxic therapies, but there is considerable disagreement in the literature over their specificities for Gli1 or Gli2. In order to develop useful, target specific Gli inhibitors, the field must make a distinction between the transcriptional targets and roles of Gli1 and Gli2. Furthermore, these transcription factors are themselves regulated (or dysregulated) by a complex network of protein-protein interactions. Thus, the future of Hh-targeted therapies lies in the ability to identify and elucidate the molecular mechanisms which enable cancers to resist treatment.
